# Augmenting Household Expenditure Forecasts with Online Employee-generated Company Reviews

**DOI:** 10.1093/poq/nfab017

**Published:** 2021-09-01

**Authors:** Efthymia Symitsi, Panagiotis Stamolampros, Antonios Karatzas

## Abstract

We assess the ability of online employee-generated content in predicting consumption expenditures. In so doing, we aggregate millions of employee expectations for the next six-month business outlook of their employer and build an employee sentiment index. We test whether forward-looking employee sentiment can contribute to baseline models when forecasting aggregate consumption in the United States and compare its performance to well-established, survey-based consumer sentiment indexes. We reveal that online employee opinions have incremental information that can be used to augment the accuracy of consumption forecasting models and inform economic policy decisions.

## Introduction

Consumer spending is a key engine that drives economic growth accounting for almost 60 percent of the Gross Domestic Product worldwide (World Bank 2020). Therefore, policymakers and practitioners closely monitor and attempt to accurately predict changes in private consumption, since these have profound effects on individual firms, sectors, and the overall economy (Fornell, Rust, and Dekimpe 2010). Forecasting private consumption has also attracted academic interest; a long research tradition has focused on how private consumption is associated with macroeconomic variables (e.g., inflation and unemployment rates), and how it responds to fiscal interventions (Katona 1971; Cogoy 1995; Hjelm 2002; Linnemann 2006).

The predictive ability of consumer sentiment indexes is central to this research stream (Ludvigson 2004; Lahiri, Monokroussos, and Zhao 2016; Barnes and Olivei 2017). Survey-based consumer indicators are widely used and offer informational value (Ludvigson 2004). For example, Carroll, Fuhrer, and Wilcox (1994) and Bram and Ludvigson (1998) show that these indexes improve consumer spending forecasts in the United States, while similar results are reported in other countries (Easaw, Garratt, and Heravi 2005; Dreger and Kholodilin 2013). This is in line with the wisdom-of-the-crowd concept, which posits that aggregated opinions of a group of individuals are more informative than the opinions of separate individuals, even if the latter are domain experts (Da and Huang 2020).

The recent explosion of online platforms allows practitioners and academics to enrich forecasting models with data generated online.^1^ Augmenting “traditional” demand forecasting methods with online user-generated content has created fruitful research directions (Chong et al. 2016; Cui et al. 2018; Lau, Zhang, and Xu 2018). This is based on the premise that incorporating human judgment in standard quantitative models, known as judgmental forecasting, increases forecasting power (Arvan et al. 2019). Online platforms designated especially for employees, such as *Glassdoor*, constitute a novel case of electronic word of mouth, allowing users to share their opinions about their employers. Unsurprisingly, this source of data attracts increasing academic interest. For example, in finance, employee satisfaction ratings have been found to predict firm performance (Huang et al. 2015; Symitsi, Stamolampros, and Daskalakis 2018; Green et al. 2019). An additional and promising piece of information shared by employees is their expectations of the six-month-ahead business outlook of their employer, a data source that has been scarcely examined in forecasting applications despite its forward-looking nature (Hales, Moon, and Swenson 2018; Huang, Li, and Markov 2020). In this work, we argue that those employee expectations capture individuals’ future labor income uncertainty, with direct implications for their willingness to spend. This is in line with economic theory, which suggests that a change in perceived future labor income uncertainty translates into a change in purchasing behavior, including the level of spending (Friedman 1957). It is also consistent with bottom-up, behavioral macro-economic models (De Grauwe 2010), whereby “amateur” individual agents like employees are, due to cognitive limitations, more capable of understanding local bits of information (relating to their employer), and use simple rules of behavior (when deciding on their personal spending). Hence, an aggregated measure across all employees, firms, and sectors may have predictive ability when forecasting changes in macroeconomic indicators, such as private consumption.

The purpose of this study is to examine whether an index that aggregates employee expectations for the near-term business outlook of their employer, named *Employee Sentiment (ES)*, predicts changes in private consumption. We perform a comprehensive empirical analysis comparing it to two well-established, leading economic indicators (Curtin 2019), the *University of Michigan Consumer Sentiment Index (MCI)* and the *Conference Board Consumer Confidence Index (CCI)*. The *ES* is based on opinions that arrive voluntarily and anonymously from hundreds of employees each month, from companies across all industries of the economy. As such, it escapes the costs involved in designing and conducting high-quality surveys, while it is also of much higher frequency than would be realistic for any survey instrument aiming to measure a specific phenomenon repeatedly (Schober et al. 2016).

This study extends the literature concerned with producing valuable insights from harnessing online information. In particular, we showcase a practical application of big data assisting in consumption forecasting. We extend the literature that examines the forecasting power of social media and user-generated content by demonstrating that employee information on job listing platforms can inform macro-economic forecasting and policymaking. We also contribute to the nascent research stream that evaluates potential insights drawn from employee opinions shared online, by showing that an aggregated index possesses incremental power in forecasting private consumption.

The rest of the paper is organized as follows. Section 2 provides an overview of the relevant literature. Section 3 describes the data, the construction methodology of the Employee Sentiment measure, and the empirical methods. Section 4 presents the findings, and Section 5 discusses the implications of this study, its limitations, and avenues for future research.

## The Forecasting Ability of Online-Generated Content

Data volume and availability of online user-generated content have spurred a strong research interest in the potential of online content for forecasting purposes. A burgeoning stream of literature across various disciplines (finance, political science, marketing, and health science) explores the predictive ability of web search traffic, online reviews, blogs, social networks, and forums (e.g., Antweiler and Frank 2004; Williams and Gulati 2008; Da, Engelberg, and Gao 2011; Charles-Smith et al. 2015). Related to our work, several empirical studies investigate online user-generated information in forecasting product and service demand, providing evidence that models at various levels of analysis (product, firm, overall economy), augmented with such information, have increased predictive ability (Chong et al. 2016; Cui et al. 2018; Schaer, Kourentzes, and Fildes 2019).

When it comes to consumer goods demand, Cui et al. (2018) document a significant improvement in sales forecasts of an apparel retailer after considering interactions between *Facebook* users, while Fantazzini and Toktamysova (2015) display the superiority of models that incorporated *Google* search data when forecasting monthly car sales. Chong et al. (2016) find that interactions among *Amazon.com* reviews, sentiment, and online marketing promotional strategies are important predictors of product sales. Bughin (2015) shows that models augmented with social media valence (*Twitter, Facebook*, blogs) improve sales forecasts. Examples of studies displaying considerable gains after incorporating online data in demand forecast models for particular Stock-Keeping-Units are Boone et al. (2018) and Schneider and Gupta (2016).

Similarly, for services, Choi and Varian (2012) show that a *Google Trends* index improves the forecasting accuracy of tourist arrival models, while the composite search index of Li et al. (2017) outperforms various benchmarks when estimating tourist visits in Beijing. Kulkarni, Kannan, and Moe (2012) show that online searches enhance the predictions for the opening-week sales of movies, while there exist empirical works that use employee online reviews to predict hotel occupancy (Viglia, Minazzi, and Buhalis 2016).

Departing from this literature, our interest lies in online content generated by a certain type of users (i.e., employees), and its potential to predict macro-level private consumption.

### The Potential of Employee-Generated Online Content

The proposed index of *Employee Sentiment* (*ES*) accumulates employee expectations of their employer’s business outlook. We posit that the aggregation of these employee expectations will provide incremental power as a predictor of private consumption. In a nutshell, our argument is built upon two premises: a) an employee’s expectation regarding the future business outlook of their employer will be more than just an uninformed guess; and b) given that all employees are, invariably, also consumers of products and services, they will adjust their consumption behavior upon those expectations.

###  

*Employees as processors of firm-specific information.* Employees are conduits and processors of all sorts of information pertinent to the conditions that their company is facing, and of factors that are determinative for the performance of their team, department, and by extension their firm (Kogut and Zander 1992; Nonaka 1994). For example, they receive and act upon information relating to product and process quality issues, internal budget expansions (or contractions), salary increases and bonuses, supply shortages, order volume changes by key customers, and so on. Moreover, through personal interaction, they become witnesses to the emotional displays and affective states of their co-workers and managers; as such, they can formulate a reasoned assessment of the firm’s organizational climate, a determinant of financial performance (Burton, Lauridsen, and Obel 2004).

It is reasonable to assume, then, that if asked to judge the business prospects of their employer, employees weigh all the available information and arrive at an informed expectation. The advent of job listing websites, such as *Glassdoor*, that allow employee-generated content, means that such information ceases to be private knowledge. Previous research argues that the voluntary and anonymous nature of employee online reviews addresses several limitations of internal informative processes, offering complementary information to firms (Symitsi et al. 2021). As such, publicly shared employee expectations about the future prospects of their employer serve as an additional disclosure channel for a firm.

A key assumption of this work, which is incorporated in the construction of the index, is that both high-level managers as well as rank-and-file employees possess valuable internal information to form well-grounded expectations. This intuition is supported by studies showing that stock option exercises of senior staff are no more informative than those of junior employees (Huddart and Lang 2003; Babenko and Sen 2015), and by Huang, Li, and Markov (2020) specifically, who find that the accuracy of firm profitability forecasts increases with the number and diversity of employee predictions. Besides, it has been argued that aggregating over a large crowd can ensure that individual errors “cancel out” insofar as they are not systematically correlated (Subrahmanyam and Titman 1999; Huang 2018). Empirical analyses have, in fact, reported incremental informational value of employee online reviews for predicting firm fundamentals and stock price changes (Symitsi, Stamolampros, and Daskalakis 2018; Green et al. 2019). In the same spirit, Hales, Moon, and Swenson (2018) find that firm-level business outlook expectations of employees posted on *Glassdoor* are good predictors of firm-level future sales, gross margin, operating income, and income before extraordinary items.

###  

*Confidence in employers and its effect on consumption expenditure.* It is generally accepted that most consumers focus predominantly on the economic conditions they personally face, rather than macro-economic conditions (Curtin 2019). We argue that an employee’s expectation of their employer’s future business performance and growth will have direct implications for the individual’s perceived probability of losing their job (and distribution of compensation in the case of redundancy), as well as the distribution of future wages (including bonuses) conditional on remaining employed by the firm. Undoubtedly, these elements affect the expectation of future income (un)certainty (Guiso, Jappelli, and Pistaferri 2002), and consequent willingness to purchase goods and services.

The linkage between income uncertainty and (household) consumption has been extensively studied in the economics literature. The “Life Cycle and Permanent Income” hypothesis posits that current consumption is affected by the discounted value of future income. A central implication is that household consumption should respond less to the expected aggregate income (or predictable changes in it) (see West 1988; Campbell and Deaton 1989) and more to the uncertainty surrounding future income. Specifically, the commonly called “Buffer-stock” models suggest that individuals facing greater income uncertainty consume less (Carroll 1994); “prudent” or risk averse consumers choose to save more, due to precautionary motives (Deaton 1991; Ben-David et al. 2018). In a recent work, Alfaro and Park (2020) match micro-data from financial accounts of US households to firms listed on the US stock exchange, and provide novel evidence that households reduce their monthly consumption in response to increases in uncertainty regarding their employer (measured as forward-looking option-implied volatility). Similarly, we argue that changes in employee expectations of their employers’ business outlook imply changes in their labor income, which we anticipate affecting their consumption behavior.

Beyond firm-level outcomes, aggregated measures of subjective judgments of economic actors have been shown to have predictive power at the macro level. For example, Fornell, Rust, and Dekimpe (2010) find that aggregated changes in customer satisfaction explain 23 percent of the variation in one-quarter-ahead growth in consumer spending. We argue that this will also be the case for an aggregated measure that captures the expected business outlooks of various firms across all sectors, as perceived collectively by employees. Using this *ES* measure, we test whether it can predict the state of the economy, and thus detect changes in overall consumption. To some extent, our approach resembles that followed by widely established, survey-based indexes. For instance, the expectation components of the *MCI* and *CCI* are constructed by combining questions, some of which ask participants to provide their opinion about the economy and the business conditions for the next 12 months and five years for the former, and the next six months for the latter (Linden 1982). As detailed in the following section, the information used to build our index is based on a question about the business outlook of one’s employer for the next six months.

## Data and Methodology

### Employee Sentiment

We construct *Employee Sentiment* (*ES*) using online employee reviews from *Glassdoor*.^2^*Glassdoor* is an online recruiting platform that encourages employees to post employer reviews. Employees can access employer information under a “give-to-get model” (Marinescu et al. 2018). This means that they should complete an anonymous review for a current or former employer for unlimited access to the content of the site for one year, including company reviews, salary information, and interview questions. Then, access to the platform is renewed with an updated review, though only one review contribution per year per company per review type (company, salary, benefit, interview, etc.) is permitted, ensuring that no multiple reviews come from the same person for the same company. Marinescu et al. (2018) find that this policy reduces polarization (only extremely positive or negative reviews) by encouraging employees with moderate views to provide employer feedback. *Glassdoor* has established mechanisms to verify users and identify fake reviews or reviews incentivized by companies and ensure reviewer anonymity. Altogether, *Glassdoor* has created an online community that allows employees’ voices to be heard offering valuable inside information for various work aspects.

More specifically, employees are encouraged to anonymously rate their employer on overall satisfaction, career opportunities, compensation and benefits, work-life balance, culture and values, management, CEO, and business outlook. The *ES* index uses the business outlook rating (enabled after May 2012), allowing employees to evaluate the six-month-ahead prospects of their employer as “Better,” “Same,” or “Worse.” This information resembles the information in widely applied survey-based consumer sentiment indexes (*MCI, CCI*) (see Bram and Ludvigson 1998; Ludvigson 2004). For example, one of the Michigan Survey of Consumer Sentiment questions asks 500 consumers each month to predict “Good,” “Uncertain,” or “Bad” business conditions in the country for the next 12 months. The respective question in the monthly Conference Board Survey of Consumer Confidence asks 5,000 consumers to predict whether the following six-month business conditions will be “Better,” “Same,” or “Worse.” Both indicators aggregate individual predictions based on bull-bear spread methodologies, which are well-established practices in measuring sentiment (Brown and Cliff 2005).

Our initial sample consists of 5,893,363 reviews from current and former employees from all organizations on *Glassdoor*. We retain only reviews from current employees, resulting in a sample of 2,778,343 reviews from June 2012 to July 2018. This ensures that the *ES* will not be driven by dissatisfied former employees (Symitsi, Stamolampros, and Daskalakis 2018). An additional reason for this filter is that former employees’ predictions of the near-term outlook might be outdated and inaccurate (Green et al. 2019). Since business outlook is an optional criterion, all reviews with missing values are removed.^3^ Hence, our final sample includes 2,256,735 reviews.

Out of this sample, 59 percent of the reviewers consider that their employers have a positive business outlook, 18 percent a negative one, and 23 percent a neutral. The Spearman correlation between the overall satisfaction and business outlook is ρ = 0.69. A positive correlation between the two rating aspects is expected, as companies with a better business outlook may provide better conditions for their employees; employees of such companies might also have a higher sense of job security (Origo and Pagani 2009).^4^

It is worth noting that our sample of employees who post on *Glassdoor* is not representative of the entire population of consumers (for instance, under-16s and retirees). Moreover, it is possible that the sample is not balanced between white-collar and blue-collar workers or between larger and smaller companies, to reflect the equivalent proportions in the labor market. Nevertheless, *Glassdoor* covers a fraction of consumers with strong purchasing power and disposable income, that is, educated, full-time, white-collar workers of large companies. As argued earlier, those employees’ informed beliefs on their future labor income uncertainty will affect their willingness to buy, and will result in an adjustment of their spending behavior. Furthermore, as consumers, these employees have arguably the highest “ability to buy,” due to a high salary and disposable income, which, based on their expectations, is distributed among consumption, savings, and investments. As such, we argue that their spending behavior will have, in relative terms, the largest bearing on aggregate public spending. Hence, despite a potential lack of population representativeness, our limited focus on *Glassdoor* employees likely achieves “topic coverage” (Schober et al. 2016). Equivalent to “opinion formers” or “elite communicators” (Ampofo, Anstead, and O’Loughlin 2011; Schober et al. 2016), who can represent the view of the broader public regarding a social issue, employees posting on platforms such as *Glassdoor* can be considered as “elite consumers.” Following Schober et al. (2016), online employee posts may capture the population-wide distribution of behaviors relevant to the topic (i.e., private consumption), even though those consumers’ characteristics do not reflect the characteristics of the full population. Consequently, we expect the predictions based on the *ES* index to be comparable with those of survey indicators, such as the *MCI* and *CCI*.

A monthly aggregate measure of employee sentiment is constructed following a two-step process: For each month *t* and every reviewed company *i*, the average firm outlook, $\hat{\text{BO}}$, is computed as follows:

(1) $${\text{BO}}_{it}=\frac{\sum ({\text{Business}\text{\textbackslash{} Outlook}}_{it}==“\text{Better}”-{\text{Business}\text{\textbackslash{} Outlook}}_{it}==“\text{Worse}”)}{N_{it}},$$

where *N_it_* is the total number of reviews in month *t* for company *i*. Then, the *Employee Sentiment, ES*, for every month *t* is derived by averaging the$\hat{\text{BO}}$ for all firms:

(2) $${\text{ES}}_{t}=100\sum {\overset{}{\text{BO}}}_{it}/M_{t},$$

where *M_t_* is the total number of companies for month *t*.

An important advantage of this data is that *ES* could also be built by sector. The Bureau of Economic Analysis reports consumption expenditures separately for services, durables, and nondurable goods. For those categories, as supplementary analysis, we examine three variants of the index where we take into account only reviews for companies that belong to respective sectors based on the methodology described in the Bureau of Economic Analysis.^5^

The aggregate *ES* has several appealing properties. The two-step construction methodology allows an equal representation of all companies in the sample irrespective of their characteristics. As a result, the *ES* captures not only the sentiment in public or large firms, but also the sentiment in thousands of small-size private companies; only 32 percent of the total sample comes from employees of publicly listed firms. Moreover, an indicator drawing from a large number of companies irons out idiosyncratic employee sentiment errors from biased predictions, which might arise from the relationship of an employee with their employer, or the particular conditions in “outlying” firms that are considerably different to the wider population. These predictions come indiscriminately from all industries, making the *ES* a well-representative aggregated proxy. Hence, every month an average (min., st.dev., max.) of 30,500 (5,388, 13,487, 53,833) business outlook predictions arrive from an average (min., st.dev., max.) of 13,860 (3,331, 5,661, 22,951) organizations over the tested period. Increasing participation from employees per month is justified by the increasing popularity of the platform.

Research using online data, such as online reviews, or opinions taken from social media platforms, may raise ethical concerns about data collection, storage, and analysis and must ensure that it respects the privacy, ownership, consent, security, and confidentiality of participants (Townsend and Wallace 2016; Humphreys and Wang 2018; Taylor and Pagliari 2018). This research complies with such principles. Data was not gathered via online scrapping methods but was directly shared by *Glassdoor* under a strict confidentiality agreement.^6^ Based on a minimization review, only the variables needed for this empirical study were accessed. Therefore, personal identifiers are not part of this dataset. Because re-identification could only be possible in extremely rare cases from metadata (e.g., reviews from unique job roles or companies with a small number of employees) and under *Glassdoor*’s terms, our raw dataset is securely stored. Moreover, the aggregated rather than individualized nature of our analysis makes the identification of reviewers from our published output impossible. With regard to informed consent of online users, we do not have an explicitly stated consent, but the permission is indirectly granted through the terms users have agreed upon for using the platform (which include *Glassdoor* sharing the data with third parties for data analysis and research purposes). In sum, our analysis uses only information that users have agreed to share.

### Benchmark Sentiment Indicators

The predictive power of *ES* is compared with that of two prominent survey-based indicators: the *University of Michigan Consumer Sentiment Index (MCI)* and the *Conference Board Consumer Confidence Index (CCI)*. To increase comparability, for both survey indexes the expectation components are used; that is, the indexes estimated are based exclusively on forward-looking questions rather than the total number of questions.^7^ Moreover, as reported in the literature, the expectation indexes display greater forecasting power than the present condition indexes (Bram and Ludvigson 1998; Ludvigson 2004). The expectation components of the *MCI* and *CCI* are taken from *Thomson Reuters Eikon*.

The proposed *ES* indicator has several advantages compared to the *MCI* and the *CCI*. First, these survey-based indicators are restricted to a limited sample of participants, while *ES* uses online information arriving from millions of employees from thousands of companies across all sectors. Second, employees express their expectations about the business outlook of their employers, while participants in the *MCI* and *CCI* surveys are asked, besides their own family conditions, to predict overall business and market conditions. Therefore, by aggregating employee opinions formed by up-to-date internal knowledge about their employers rather than the overall economy, *ES* is based exclusively on individuals’ immediate experience. Third, in addition to market and firm-level indicators, industry-specific indicators can be constructed to reflect the employee sentiment in specific industries which, in turn, could be useful for detecting significant sector-specific changes in demand.

**Figure 1.**
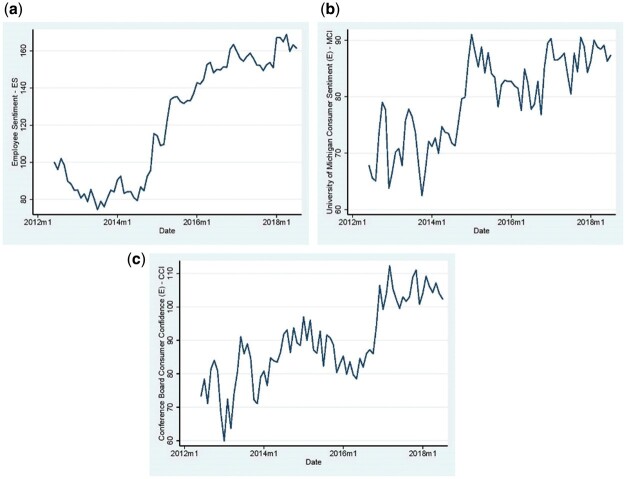
**Employee Sentiment and Consumer Sentiment indexes.** Panel a displays the Employee Sentiment aggregating online opinions from employees in the United States who voluntarily and anonymously disclose their expectations for the business outlook of their employer for the next six months. Panels b and c show the University of Michigan and the Conference Board Consumer indicators. The sample spans the period from June 2012 to July 2018 .

Figure 1 shows the *ES* and the consumer sentiment indicators graphically. Overall, the study period is marked with an upward trend in the level of employee and consumer sentiment. The raw values of the *ES* display a significant and positive correlation with the *MCI* and the *CCI* of 0.74 and 0.54, respectively, while the correlation between the *MCI* and the *CCI* is 0.72.

Following Vosen and Schmidt (2011), we take changes in sentiment rather than levels (monthly year-on-year growths). Using changes ensures that the results are comparable across the benchmarks and robust to differences in the construction methodologies, starting years, and seasonality (Bram and Ludvigson 1998; Ludvigson 2004). This also mitigates multicollinearity concerns, allowing us to test models enriched with all indexes together to investigate whether the information content of the *ES* is subsumed by the other proxies or carries complementary information.^8^ Changes in the *ES* are only weakly correlated with changes in the benchmark sentiment indexes.

### Consumption Expenditures

The variables to be forecast are the monthly year-on-year natural logarithmic differences (growth) of four real household consumption spending types, Δ*ln*(*C_t_*), namely, the total personal consumption expenditure (PCEC), the durable goods personal consumption expenditure (PCEDG), the nondurable goods personal consumption expenditure (PCEND), and the services personal consumption expenditure (PCESC), taken from the *Federal Reserve Bank of St. Louis (FRED)*.

### Additional Variables

In line with prior research (Bram and Ludvigson 1998; Vosen and Schmidt 2011), we control for the real US stock price measured by the S&P500 index, *S&P500defl*, the real personal income, *PIdefl*, and the three-month US Treasury bill rate, *TBL* (all variables are in year-on-year growths). The stock market prices and the Treasury bill rate are taken from *Thomson Reuters Eikon*. The personal income measures the wages and salaries plus transfers minus personal contributions for social insurance, sourced from *FRED*. The real values are estimated using the implicit price deflator for personal consumption expenditures from *FRED*. Table 1 displays key descriptive statistics of the variables.

**Table 1.** Descriptive statistics

	Description	Mean	Std. dev.	Min	Max
ES	Employee Sentiment	11.233	17.308	−25.696	53.367
MCI	University of Michigan Consumer Sentiment (Expectations)	4.312	10.083	−23.428	24.537
CCI	Conference Board Consumer Confidence (Expectations)	6.895	13.127	−15.138	29.930
PCEC	Total Personal Consumption Expenditures	2.740	0.728	1.265	4.358
PCEDG	Durable Goods Consumption Expenditures	6.414	1.634	1.066	11.044
PCEND	Nondurable Goods Consumption Expenditures	2.601	0.715	0.915	3.943
PCESC	Services Consumption Expenditures	2.212	0.794	0.158	3.851
S&P500defl	Real S&P500 Prices	10.196	7.136	−9.286	24.479
PIdefl	Real Personal Income	2.660	1.692	−3.280	5.616
TBL	3-month US Treasury-bill Rate	0.271	0.355	−0.090	1.050

Note.—This table presents key descriptive statistics of the variables used in the empirical part. All variables are expressed in monthly year-on-year logarithmic differences.

### Models and Methods

The empirical analysis investigates the ability of the *ES* to forecast consumption. In so doing, we perform both in-sample (*IS*) and out-of-sample (*OOS*) analyses following the methodology of Vosen and Schmidt (2011). *IS* uses all the sample (June 2012–July 2018) to estimate the model parameters and then makes one-step-ahead forecasts. *OOS* withholds a smaller sample of the observations (window) to estimate the model parameters and then obtains a one-step-ahead forecast beyond those in the estimation sample (like real-world forecasting applications). The first sample starts from June 2012 to December 2015 (window of 42 observations). This process is repeated by adding one forward observation to the sample, estimating new model parameters, and obtaining the one-step-ahead forecast until we reach the end of the sample. This process gives us a time series of forecasts (Hyndman 2006).^9^ As argued in the forecasting literature, a model with good in-sample performance does not necessarily work equally well in the real-world forecasting environment predicting truly unseen values (Tashman 2000; Rapach and Wohar 2006).

The baseline model (*B*_0_) is a simple autoregressive model of consumption growth augmented with macroeconomic variables, which are typically used in the extant literature (Carroll, Fuhrer,and Wilcox 1994), described as follows:

(3) $$\Delta ln(C_{t:t+1})=\alpha (L)\Delta \ln(C_{t-j})+\delta (L)Z_{t-j}+\epsilon_{t:t+1},$$

where Δ*ln*(*C_t_*_:__*t*__+1_) is the monthly year-to-year growth rates of consumption expenditures, *Z_t_* controls for year-to-year growths of the real US stock price, the real personal income, and the three-month US Treasury bill rate. The optimal number of lags, *j*, is determined based on the Schwarz information criterion (up to a maximum of 3 lags). The error term, *s_t_*_:__*t*__+1_, is assumed to follow a first-order moving average process, MA(1) (Bram and Ludvigson 1998; Vosen and Schmidt 2011).

We then examine the predictive ability of the *ES* and additional sentiment measures with the following models:

(4) $$\Delta ln\left(C_{t:t+1}\right)=\alpha \left(L\right)\Delta \ln\left(C_{t-j}\right)+\beta \left(L\right)\Delta \ln\left(S_{t-j}\right)+\delta \left(L\right)Z_{t-j}+\epsilon_{t:t+1},$$

where *S_t_* takes value from monthly year-to-year growths of the *ES* (*M*_1_), *MCI* (*M*_2_), or the *CCI* (*M*_3_).

To test whether the sentiment measures statistically improve IS and OOS predictions in household expenditure, the mean squared forecast errors (MSFE) of the augmented models with the sentiment proxies (M1-M3) are compared with those of the B_0_ using the adjusted-MSFE method developed by Diebold and Mariano (1995) and West (1996) and corrected by Clark and West (2007). We also compare directly the *ES* measure with the alternative sentiment benchmarks (*M*_1_ versus *M*_2_ and *M*_3_) using the Diebold and Mariano (1995) statistic (see Appendix A for a description).

Finally, we employ an extended baseline model (*B*_1_) that includes all the sentiment benchmarks, described as follows:

(5) $$\Delta \ln\left(C_{t:t+1}\right)=\alpha \left(L\right)\Delta ln\left(C_{t-j}\right)+\beta_{1}\left(L\right)\Delta ln\left(\mathrm{MC}I_{t-j}\right)+\beta_{2}(L)\Delta ln(\mathrm{CC}I_{t-j})+\delta (L)Z_{t-j}+\epsilon_{t:t+1},$$

We then test whether the inclusion of the *ES* in the *B*_1_ (model *M*_4_) offers significant benefits in predicting consumption expenditures.^10^

## Empirical Analysis

### Aggregate Consumption and In-Sample Predictive Ability of Employee Sentiment

Table 2 presents the in-sample (*IS*) results revealing the predictive power of the *ES* and benchmark indicators over the entire sample period. Columns (1)–(3) report the ratio of root mean squared forecast errors, *RMSFE*, from models *M*_1_ to *M*_3_ over the *RMSFE* from the baseline model, *B*_0_. Statistically significantly less-than-unit values exhibit that sentiment indexes added to *B*_0_ improve the accuracy of the parsimonious model in predicting consumption expenditures and reducing forecast errors. If the baseline model is found to produce on average smaller forecast errors compared to the proposed model (above-than-unit RMSFE ratios), we also report whether the differences are statistically significant and, thus, whether the baseline model is better than the proposed model (p-values in parentheses for one-side tests).

**Table 2.** In-sample predictive ability as indicated by root mean squared forecast error ratios (*p*-values in parentheses)

Outcome	*ES:M_1_/B_0_*	*MCI:M_2_/B_0_*	*CCI:M_3_/B_0_*	*ES/MCI*	*ES/CCI*	*ES:M_4_/B_1_*
	(1)	(2)	(3)	(4)	(5)	(6)
PCEC	0.848	0.959	0.977	0.885	0.868	0.885
	(0.000)	(0.005)	(0.024)	(0.075)	(0.050)	(0.002)
PCEDG	0.919	0.989	0.966	0.929	0.951	0.929
	(0.009)	(0.152)	(0.014)	(0.134)	(0.197)	(0.005)
PCEND	0.960	0.946	0.920	1.015	1.044	0.955
	(0.036)	(0.032)	(0.005)	(0.131)	(0.278)	(0.010)
PCESC	0.967	1.035	1.022	0.935	0.946	0.975
	(0.042)	(0.075)	(0.052)	(0.238)	(0.292)	(0.047)

Note.—This table presents the in-sample power of changes in the *Employee Sentiment* indicator (*ES*), the University of Michigan Consumer Sentiment index (*MCI*), and the Conference Board Consumer Confidence index (*CCI*) for one-step-ahead forecasts of consumption growths (PCEC, PCEDG, PCEND, PCESC for total, durable goods, nondurable goods, and service consumption, respectively). Columns (1)–(3) display the ratio of root mean squared forecast errors (RMSFE) from models (*M_1_–M_3_*) over the RMSFE from the baseline model B_0_, described in Eq.(3). Columns (4)–(5) compare directly the RMSFE of the *ES* model, *M_1_*, to the benchmark models *M_2_* and *M*_3_. The last column compares a model that includes all the sentiment proxies (*M_4_*) with an alternative baseline model *B_1_*, described in Eq.(5). In all the models, the standard errors are assumed to follow a moving average (MA(1)) process. P-values reported in parentheses denote the level of significance for one-side tests from the Diebold and Mariano (1995) and West (1996) test corrected by Clark and West (2007) for the nested models and the Diebold and Mariano (1995) for non-nested models, which evaluate statistically the performance of the models.

The results show that the baseline *B*_0_ augmented by the *ES* significantly improves the predictive accuracy for all consumption expenditures. The survey-based indicators have also significant benefits in predicting consumption expenditures in most cases, but they both underperform compared to *B*_0_ in predicting services consumption expenditures (*M*_2_–*M*_3_ vs. *B*_0_).

Columns (4)–(5) of table 2 compare directly the *IS* predictive power of the *ES* to that of the benchmark indicators (*M*_1_ vs. *M*_2_–*M*_3_). Despite that the *ES* forecasts have less noticeable differences in statistical terms to those of the alternative indexes, we find that the *ES* offers statistically significant and complementary benefits beyond the consumer sentiment proxies altogether (*M*_4_ vs. *B*_1_; Column (6)), indicating that it carries unique information.

### Predicting Aggregate Consumption Out-of-Sample with Employee Sentiment

This part evaluates the out-of-sample (*OOS*) predictive power of the *ES* in table 3. *ES* adds significantly in predicting changes in total, nondurable, and services consumer spending against *B_0_*. The *MCI* contains only marginally superior forecasting power for services consumption expenditures, while the *CCI* significantly deteriorates the forecasts compared to *B_0_*.

**Table 3.** Out-of-sample predictive ability as indicated by root mean squared forecast error ratios (*p*-values in parentheses)

Outcome	*ES:M_1_/B_0_*	*MCI:M_2_/B_0_*	*CCI:M_3_/B_0_*	*ES/MCI*	*ES/CCI*	*ES:M_4_/B_1_*
	*(1)*	*(2)*	*(3)*	*(4)*	*(5)*	*(6)*
PCEC	0.980	0.972	1.049	1.008	0.935	1.004
	(0.107)	(0.171)	(0.428)	(0.450)	(0.200)	(0.354)
PCEDG	1.044	1.020	1.174	1.023	0.889	0.990
	(0.249)	(0.325)	(0.136)	(0.438)	(0.255)	(0.246)
PCEND	0.952	0.993	1.077	0.959	0.884	0.965
	(0.001)	(0.189)	(0.099)	(0.292)	(0.204)	(0.073)
PCESC	0.949	0.898	1.056	1.057	0.899	1.074
	(0.034)	(0.131)	(0.047)	(0.329)	(0.021)	(0.059)

Note.—This table presents the out-of-sample power of growths in the *Employee Sentiment* indicator (*ES*), the University of Michigan Consumer Sentiment index (*MCI*), and the Conference Board Consumer Confidence index (*CCI*) for one-step-ahead forecasts of consumption growths (PCEC, PCEDG, PCEND, PCESC for total, durable goods, nondurable goods, and service consumption, respectively). Columns 1–3 display the ratio of root mean squared forecast errors (RMSFE) from models (*M_1_–M_3_*) over the RMSFE from the baseline model *B_0_* (Eq. 3). Columns 4–5 compare directly the RMSFE of the *ES* model, *M_1_*, to the benchmark sentiment models *M_2_* and *M_3_*. The last column compares a model that includes all the sentiment proxies (*M_4_*) with an alternative baseline model *B_1_* (Eq. 5). In all the models, the standard errors are assumed to follow a moving average (MA(1)) process. *P*-values in parentheses denote the level of significance for one-side tests from the Diebold and Mariano (1995) and West (1996) test corrected by Clark and West (2007) for the nested models and the Diebold and Mariano (1995) for non-nested models, which evaluate statistically the performance of the models.

When we test the *OOS* performance of the *ES* against the *MCI* (Column (4)), the former has no significant differences in reducing the forecast errors. Compared to the *CCI* (Column (5)), the *ES* generates smaller forecast errors in all cases, though the differences are only significant in services consumer spending. In an extended model, including all sentiment indexes, our findings regarding the information content of employee expectations are mixed (*M*_4_ vs. *B*_1_; Column (6)); the *ES* complements the information content of consumer sentiment proxies in predicting nondurable goods consumption, but in the case of services consumption, the forecasts deteriorate. While in the in-sample setting, the *ES* added value in both parsimonious and augmented baseline models, the out-of-sample setting documents better performance when the *ES* is added to a parsimonious forecasting model of consumption.

### Further Evidence on the Value of Employee-Generated Data in Forecasting Aggregate Consumption

We further explore the value of employee information in forecasting aggregate consumption in two ways. First, we build sector-specific *ES* indexes using reviews from durable goods producers, nondurable goods producers, and service firms, and examine their performance in predicting growth in the respective consumption expenditures.

Table 4 presents the results for the in-sample and out-of-sample performance of these indicators compared to the baseline models. Overall, the predictive power of the *ES* remains qualitatively similar. Even though we do not compare the industry-specific *ES* indexes to the overall *ES*, we find that aggregating the expectations of staff employed only within these industries does not offer a greater advantage in predicting private consumption than the entire sample of employees.

**Table 4.** IS and *OOS* predictive ability as indicated by root mean squared forecast error ratios (*p*-values in parentheses): industry-specific employee sentiment

	IS		OOS	
Outcome	*ES:M_1_/B_0_*	*ES:M_4_/B_1_*	*ES:M_1_/B_0_*	*ES:M_4_/B_1_*
PCEDG	0.947	0.915	1.074	0.978
	(0.034)	(0.005)	(0.155)	(0.118)
PCEND	0.956	0.953	0.885	0.931
	(0.027)	(0.011)	(0.002)	(0.031)
PCESC	0.970	0.923	0.979	1.099
	(0.049)	(0.001)	(0.104)	(0.152)

Note.—This table compares the in-sample and out-of-sample power of *Employee Sentiment* (*ES*), which considers only reviews from employees working at firms in durable, nondurable, and services industries for predicting consumption growths for durable goods, nondurable goods, and services, respectively, against two baseline models (M_1_ vs. B_0_ and M_4_ vs. B_1_) by estimating the ratio of their root mean squared errors. Columns (2) and (4) indicate the incremental predictive ability of the *ES* beyond other sentiment benchmarks, including the University of Michigan Consumer Sentiment index (*MCI*) and the Conference Board Consumer Confidence index (*CCI*). *P*-values in parentheses denote the level of significance for one-side tests from the Diebold and Mariano (1995) and West (1996) test corrected by Clark and West (2007).

Second, we examine whether the informational value and relevance of employee business outlook predictions vary with the employees’ role in the firm, constructing an alternative *ES* that uses business outlook predictions from managerial staff or staff employed in supply chain, production, accounting, or sales roles.^11^ This would suggest information and knowledge asymmetries within firms. For example, previous research has shown that information asymmetry exists between managers and rank-and-file employees, whereby the opinions of the latter group are only partially materialized in the expectations of the former (Huang, Li, and Markov 2018), while other findings in the literature dispute such asymmetries (Huddart and Lang 2003; Babenko and Sen 2015).

Table 5 presents the *IS* and *OOS* results. The findings provide evidence that opinions of employees that are not in direct contact with customers, suppliers, or supply chain and production planning are relevant, suggesting that the information content of all employees collectively is valuable.

**Table 5.** IS and *OOS* predictive ability as indicated by root mean squared forecast error ratios (*p*-values in parentheses): employee sentiment and access to superior information

	*IS*		*OOS*	
	*ES:M_1_/B_0_*	*ES:M_4_/B_1_*	*ES:M_1_/B_0_*	*ES:M_4_/B_1_*
Panel A: Staff with access to superior information				
PCEC	0.868	0.988	0.940	1.039
	(0.000)	(0.121)	(0.044)	(0.287)
PCEDG	0.968	0.970	1.136	1.055
	(0.038)	(0.029)	(0.459)	(0.365)
PCEND	0.981	0.976	0.937	0.977
	(0.106)	(0.060)	(0.002)	(0.155)
PCESC	0.986	0.987	0.971	1.070
	(0.125)	(0.123)	(0.060)	(0.267)
Panel B: Other staff				
PCEC	0.846	0.955	0.926	0.869
	(0.000)	(0.004)	(0.002)	(0.005)
PCEDG	0.957	0.966	1.080	1.090
	(0.014)	(0.007)	(0.177)	(0.095)
PCEND	0.957	0.955	0.878	0.916
	(0.023)	(0.010)	(0.010)	(0.010)
PCESC	1.005	0.943	1.009	1.137
	(0.029)	(0.019)	(0.414)	(0.178)

Note.—This table compares the in-sample and out-of-sample power of *Employee Sentiment* (*ES*), which considers reviews from staff with access to superior information versus other staff for predicting consumption growths (PCEDG, PCEND, PCESC for durable goods, nondurable goods, and service consumption, respectively). The table presents the ratio of root mean squared errors from the baseline models augmented with the *ES* model over the root mean squared errors from the baseline models. *P*-values in parentheses denote the level of significance for one-side tests from the Diebold and Mariano (1995) and West (1996) test corrected by Clark and West (2007).

**Table B1.** IS and OOS predictive ability as indicated by root mean squared forecast error ratios (*p*-values in parentheses): alternative employee sentiment indexes

	*IS*		*OOS*	
	*ES:M_1_/B_0_*	*ES:M_4_/B_1_*	*ES:M_1_/B_0_*	*ES:M_4_/B_1_*
Panel A: Equal-weighted index				
PCEC	0.790	0.844	0.823	0.991
	(0.000)	(0.001)	(0.001)	(0.214)
PCEDG	0.929	0.919	1.068	1.005
	(0.019)	(0.009)	(0.160)	(0.280)
PCEND	0.965	0.979	1.015	1.010
	(0.035)	(0.057)	(0.084)	(0.410)
PCESC	0.943	0.894	0.896	1.164
	(0.016)	(0.000)	(0.003)	(0.040)
Panel B: Filtered index				
PCEC	0.813	0.947	0.837	0.986
	(0.000)	(0.010)	(0.005)	(0.157)
PCEDG	0.953	0.952	1.084	1.067
	(0.022)	(0.018)	(0.325)	(0.109)
PCEND	0.990	0.996	1.037	1.003
	(0.091)	(0.294)	(0.180)	(0.398)
PCESC	0.995	0.889	0.931	1.110
	(0.036)	(0.000)	(0.016)	(0.329)

Note.—This table compares the in-sample and out-of-sample power of alternative *Employee Sentiment* (*ES*) proxies, for predicting consumption growths (PCEDG, PCEND, PCESC for durable goods, nondurable goods, and service consumption, respectively). The table presents the ratio of root mean squared errors from the baseline models augmented with the *ES* model over the root mean squared errors from the baseline models. Panel A constructs an *ES* index that weighs equally all the reviews per month (one-step process). Panel B constructs an *ES* index using only the companies with at least five reviews per month. *P*-values denote the level of significance for one-side tests from the Diebold and Mariano (1995) and West (1996) test corrected by Clark and West (2007).

### Robustness Checks

In Appendix B, we examine alternative *ES* indexes: We estimate an *ES* as a one-step process by averaging all the reviews per month, thus placing more weight on firms with a larger number of employee reviews. We also construct an index filtering out firms with less than five reviews each month, as in Green et al. (2019); therefore, firms with a small number of employees are less likely to participate in the index.

As our index is considered to manifest through future income uncertainty, we test the *ES* against a sentiment proxy that measures only income expectations.^12^ To this end, we replace the total expectations *MCI* with the University of Michigan Consumer Survey from personal finances, that is, the expected change in real income during the next year. From these analyses, the findings remain consistent with *ES* adding value to both parsimonious and augmented consumption forecasting models.

## Discussion and Conclusions

We extend the stream of research that evaluates the usefulness of novel sources of online data. In particular, we assess the informational value of data generated by an important group of stakeholders with unprecedented potential, that of employees, in forecasting private consumption. In doing this, we introduce a sentiment indicator that aggregates employee opinions of their employers’ future business outlook, shared voluntarily on *Glassdoor* ’s platform. This *Employee Sentiment* indicator is found to be a significant predictor both in-sample and out-of-sample of four types of consumer spending growth in the United States, generally adding value beyond two well-established, survey-based consumer sentiment indexes with stronger results in parsimonious consumption forecasting models.

From a research perspective, this study exhibits that external sources of information and, particularly, social media platforms can add value in forecasting applications (Vidgen, Shaw, and Grant 2017). Moreover, this work extends the literature examining the predictive power of aggregated online user-generated information (Rui, Liu, and Whinston 2013; Hu, Koh, and Reddy 2014) and, particularly, the research stream that evaluates the informational value of employees’ opinions (Huang et al. 2015; Symitsi, Stamolampros, and Daskalakis 2018). In line with the research that examines survey-based indicators in forecasting private consumption expenditures (Bram and Ludvigson 1998; Vosen and Schmidt 2011; Woo and Owen 2019), this work proposed an alternative measure that can significantly enhance aggregate demand forecasting. Our index is tested against baseline and enriched models with the *Michigan Consumer Sentiment* and the *Conference Board Consumer Confidence* expectations, extending the findings of prior research (Vosen and Schmidt 2011) and providing evidence of incremental information embedded in employee opinions.

Despite the forward-looking orientation, and similar construction methodology of all indexes, *ES* differs from the survey-based ones in three important ways. First, in asking employee-consumers to evaluate their employers’ outlook, *ES* draws from individuals’ immediate experience and personal knowledge, without implicitly assuming understanding of the entire economy (De Grauwe 2010). Second, contrary to the survey-based measures that draw from a limited number of participants per month, *ES* aggregates thousands of employee opinions from most industries. As illustrated here, this allows for forecasts based on industry-specific employee sentiment measures. Third, despite its potential lack of representativeness, employees whose opinions are incorporated in the *ES* are “elite consumers,” due to their high purchasing power and strong influence on how their household income is distributed among consumption, investing, and saving. As such, we have argued that it achieves “topic coverage” (Schober et al. 2016).

On the premise that an employee’s expectation of their employer’s business outlook reflects their uncertainty about future income, our results are aligned with insights derived from “Buffer-stock” models developed in the economics literature positing that individuals adjust their consumption in response to their expectation of how uncertain their income is. It is worth noting that in the relevant literature, scholars have devised sophisticated ways to estimate a consumer’s perceived income uncertainty or elicit one’s expectations of future income. In essence, our simple *ES* measure is a “short-cut” that can provide a continuously available and easily accessible tool to economic forecasters and policymakers.

This research is not free from limitations. First, online reviews are characterized from biases, such as a J-shaped distribution (Hu, Pavlou, and Zhang 2017), self-selection (Li and Hitt 2008), or even manipulation (Hu et al. 2012). However, such biases have been reported in customer online reviews and not in employee online reviews. Relevant literature points out that employee online reviews could be less biased (Marinescu et al. 2018; Stamolampros et al. 2019, Symitsi et al. 2021). The aggregation of employee sentiment across companies serves also in ironing out distortions coming from data manipulation and fraud by some companies. Such phenomena, though, are highly unlikely for two reasons: (a) employee accounts and reviews on *Glassdoor* are verified through systematic algorithm- and human-based controls, and (b) the reputation costs of firms that deploy such practices would exceed any benefits.

Second, the generalizability of our results requires further testing in the future. We acknowledge that this dataset is quite new, so we cannot test the behavior in long periods and different regimes (e.g., economic turbulence). With an increasing participation in such platforms, our expectation is that future research can offer additional results. We also envisage tests at a higher frequency that will be a significant advantage compared to other sentiment benchmarks that are only offered at the monthly level.

Third, many alternative macroeconomic indicators could have been considered as benchmarks. However, performing a horse race to evaluate indicators whose predictive power may vary with the context was beyond the scope of this paper (Sagaert et al. 2018). The *CCI* and *MCI* were selected because they share with *ES* the important property of capturing human sentiment as well as a very similar construction methodology. Future research can compare the *ES* with other types of macroeconomic indicators, or when forecasting additional categories of private consumption to those considered here. Moreover, as such information becomes more and more popular in other countries, there will exist opportunities for further investigation of its potential on different settings.

**Table B2.** Comparison of the *Employee Sentiment* with the expected change in real income during the next year from the University of Michigan Consumer Survey (MCI*)

	*IS*			*OOS*		
	MCI^*^:M_2_/B_0_	ES/MCI^*^	*ES:M_4_/B_1_*	MCI^*^:M_2_/B_0_	ES/MCI^*^	*ES:M_4_/B_1_*
	*(1)*	*(2)*	*(3)*	*(4)*	*(5)*	*(6)*
PCEC	0.858	0.988	0.890	0.889	1.102	0.901
	(0.000)	(0.442)	(0.000)	(0.003)	(0.093)	(0.044)
PCEDG	0.978	0.939	0.931	1.005	1.038	0.970
	(0.031)	(0.142)	(0.010)	(0.033)	(0.418)	(0.091)
PCEND	0.996	0.965	0.938	1.119	0.851	1.018
	(0.280)	(0.036)	(0.006)	(0.011)	(0.029)	(0.406)
PCESC	0.974	0.993	0.975	0.969	0.979	0.955
	(0.084)	(0.461)	(0.057)	(0.123)	(0.372)	(0.205)

Note.—This table presents the in-sample (Columns 1–3) and out-of-sample (Columns 4–6) power of growths in the *Employee Sentiment* indicator (*ES*) and the expected change in real income during the next year from the University of Michigan Consumer Survey (MCI*) for one-step-ahead forecasts of consumption growths (PCEC, PCEDG, PCEND, PCESC for total, durable goods, nondurable goods, and service consumption, respectively). Columns 1 and 4 display the ratio of root mean squared forecast errors (RMSFE) from the MCI* over the RMSFE from the baseline model B_0_ (Eq. 3). Columns 2 and 5 compare directly the RMSFE of the *ES* model to the benchmark sentiment model with MCI*. The last column compares model M_4_ that includes the *ES*, the MCI*, and the Conference Board Confidence Index (*CCI*) with an alternative baseline model B_1_ (Eq. 5). In all the models, the standard errors are assumed to follow a moving average (MA(1)) process. *P*-values in parentheses denote the level of significance for one-side tests from the Diebold and Mariano (1995) and West (1996) test corrected by Clark and West (2007) for the nested models and the Diebold and Mariano (1995) for non-nested models, which evaluate statistically the performance of the models.

## Data Availability

REPLICATION DATA are not available because of the permission policy of the original data collector. The editors have waived *POQ*’s replication policy for this manuscript. Please contact the corresponding author for more information. However, the analysis code is available at https://doi.org/10.7910/DVN/WP0PUU.

## Footnotes

1
https://www.thedrum.com/news/2018/04/17/pepsico-uses-data-science-decide-its-next-crisp-flavour-now-it-could-inform-its, accessed 2018-10-12.

2 We thank *Glassdoor* for providing us with this dataset.

3 In the review sample, 81.23 percent provides business outlook predictions.

4 We also tested whether the overall rating (ordinal scales 1 to 5) differs between reviews that post business outlook predictions versus those that do not post. The median is 4.0 for both groups. The mean overall ratings are 3.67 for the former and 3.66 for the latter. Significant differences in the rankings per employer are found only in 2.34 percent of the companies.

5
https://www.bea.gov/sites/default/files/methodologies/nipa-handbook-all-chapters.pdf#page=90

6 See https://hrtechprivacy.com/brands/glassdoor/ for details on the terms of use and privacy policy.

7 For example, for the estimation of the *MCI* expectations, three questions are considered, that is, a) Now looking ahead—do you think that a year from now you (and your family living there) will be better off financially, or worse off, or just about the same as now? b) Now turning to business conditions in the country as a whole—do you think that during the next twelve months we’ll have good times financially, or bad times, or what? c) Looking ahead, which would you say is more likely—that in the country as a whole we’ll have continuous good times during the next five years or so, or that we will have periods of widespread unemployment or depression, or what? https://data.sca.isr.umich.edu/fetchdoc.php?docid=24770, accessed 15 July 2020.

8 We are grateful to three anonymous reviewers for these suggestions.

9 A gradually expanding window is widely adopted in the literature (Bram and Ludvigson 1998) for greater parameter stability.

10 Code to replicate this analysis can be found at https://doi.org/10.7910/DVN/WP0PUU.

11
*Glassdoor* orders the job roles provided by employees to 158 broader job categories. Then, we manually classified them into: “Staff with superior access to information” and “Other staff.” Examples of such job roles are “account executive,” “accounting analyst,” “c suite,” “logistics manager,” “business analyst,” and “retail representative.” Reviews with missing values in job roles are omitted (56 percent). From the remaining reviews used for these *ES* proxies, 52 percent are classified as “superior access to information” and the remaining as “other staff.”

12 We thank an insightful reviewer for this suggestion.

## Notes

Efthymia Symitsi is an assistant professor in the Accounting and Finance Division at Leeds University Business School, Leeds, West Yorkshire, UK. Panagiotis Stamolampros is an assistant professor in the Management Division at Leeds University Business School, Leeds, West Yorkshire, UK. Antonios Karatzas is an assistant professor at Norwich Business School, University of East Anglia, Norwich, Norfolk, UK. The authors thank *Glassdoor* for providing the data for this research. We also thank three anonymous reviewers for constructive feedback, and the editors, Florian Keusch, Frederick G. Conrad, and Michael F. Schober, for helpful comments and suggestions.

## Appendix A. Statistical comparison of forecasts generated from different models

In order to compare forecasts from nested linear models, the Diebold and Mariano (1995) and West (1996) statistic, which assumes an asymptotic standard normal distribution, can be severely undersized, leading to tests with very low power. To this end, the adjusted-MSFE developed by Clark and West (2007) is employed, which accounts for the non-standard distribution found by Clark and McCracken (2001) and McCracken (2007) and has been found to perform reasonably well in terms of size and power.

As Inoue and Kilian (2005) argue, the Diebold and Mariano (1995) and West (1996) statistic is designed to also accommodate full-sample tests extending the accuracy of predictions in an in-sample analysis. Much of the subsequent research, including the adjusted statistic of Clark and West (2007), maintains the stationarity and independence assumptions that permit in-sample tests allowing one to statistically determine the performance of aggregate sentiment indicators in in-sample and out-of-sample settings. This adjusted statistic is widely applied in forecasting applications to test the null hypothesis of equal predictive accuracy (e.g., Chen 2009; Carrière-Swallow and Labbé 2013; Clark and McCracken 2013), correcting for a bias induced in the statistic when estimating the parameters in the larger model compared to a parsimonious model.

Under the null hypothesis, the expected error from the baseline model and the model that is augmented by an overall sentiment proxy is the same. Under the alternative hypothesis, the expected error from the augmented model is less than the baseline model it nests. The null (*H*_0_) and alternative (*H*_1_) hypotheses tested in this paper are as follows:

$$H_{0}:E[L_{t+1}(\theta_{0})]=E[L_{t+1}(\theta_{1})]\mathrm{vs}H_{1}:E[L_{t+1}(\theta_{0})]>E[L_{t+1}(\theta_{1})],$$

where $L_{t+1}$ denotes the squared errors and $\theta_{0}$ and $\theta_{1}$ are vectors of the parameters from the baseline model and the augmented model, respectively. If the *h*-step-ahead forecasts of $y_{t+h}$ from the baseline and the augmented models are ${\hat{y}}_{0,t:t+h}$_*h*_ and ${\hat{y}}_{1,t:t+h}$, the MSFE-adjusted statistic is computed by defining:

(A1) $$\mathrm{adj}-{\tilde{d}}_{t:t+1}={\hat{\epsilon }}_{0,t:t+1}^{2}-[{\hat{\epsilon }}_{1,t:t+1}^{2}-({\hat{y}}_{0,t:t+1}-{\hat{y}}_{1,t:t+1}{)}^{2},$$

and, subsequently, regressing the $\mathrm{adj}-\tilde{d}$ on a constant using heteroskedasticity and autocorrelation consistent (HAC) standard errors (Newey and West 1987). ${\hat{\epsilon }}_{0}^{2}$ and ${\hat{\epsilon }}_{1}^{2}$ are the forecast squared errors from the baseline and the augmented model, respectively. A p-value for a one-sided (upper-tail) test is then computed using the standard normal distribution.

The above test is applied for comparing nested models. In order to compare non-nested models (the forecasts using the *ES* vs. *MCI* or *CCI)*, we employ the Diebold and Mariano (1995) test with Newey and West (1987) HAC robust standard errors, where the null hypothesis assumes equal predictive accuracy (${\tilde{d}}_{t:t+1}={\hat{\epsilon }}_{0,t:t+1}^{2}-{\hat{\epsilon }}_{1,t:t+1}^{2}=0$). The original Diebold Mariano test used a rectangular kernel estimator of Hansen (1982); however, Newey-West HAC estimators are currently widely applied in forecasting applications (see Clark and McCracken 2013, p. 1161; Diebold 2015).

## Appendix B. Robustness checks

To increase the robustness of our results, we investigate two additional employee sentiment proxies. First, we examine the *IS* and *OOS* predictive ability of an alternative specification of the *ES* index that places more weight on firms with a larger number of employee reviews. In order to do this, we estimate this index as a one-step process, by weighting equally all the reviews that arrive every month. The results are displayed in Panel A of table B1.

Second, we construct an index that filters out firms with less than five reviews each month, following Green et al. (2019). Therefore, firms with a small number of employees are less likely to participate in the index, allowing the *ES* to be formed based on employee opinions from larger companies. The in-sample and out-of-sample predictive ability of the filtered *ES* is generally maintained. The results are found in Panel B of table B1.

Altogether, while such indexes may lessen the impact of biased responses from employers with few reviews, they also diminish the presence of employees from small businesses. In the US economy, small businesses account for 44 percent of the economic activity, hence we consider our default index as more appropriate.^13^

Finally, table B2 compares our index to an alternative component of the *MCI* from personal finances, that is, the expected change in real income during the next year, which measures only income expectations. There are still differences between the *ES* and this index, as the former focuses on the employers’ outlook, while the latter focuses on the personal outlook. Our results, generally, remain consistent with the evidence that the *ES* adds to both parsimonious and augmented consumption forecasting models.
